# Evaluating an AI Decision Support System for the Emergency Department: Retrospective Study

**DOI:** 10.2196/80448

**Published:** 2026-01-26

**Authors:** Yvette Van Der Haas, Wiesje Roskamp, Lidwina Elisabeth Maria Chang-Willems, Boudewijn van Dongen, Swetta Jansen, Annemarie de Jong, Renata Medeiros de Carvalho, Dorien Melman, Arjan van de Merwe, Marieke Bastian-Sanders, Bart Overbeek, Rogier Leendert Charles Plas, Marleen Vreeburg, Thomas van Dijk

**Affiliations:** 1 Eindhoven University of Technology Eindhoven The Netherlands; 2 St. Antonius Ziekenhuis Utrecht The Netherlands

**Keywords:** emergency department, artificial intelligence, AI, clinical impact, health care

## Abstract

**Background:**

Overcrowding in the emergency department (ED) is a growing challenge, associated with increased medical errors, longer patient stays, higher morbidity, and increased mortality rates. Artificial intelligence (AI) decision support tools have shown potential in addressing this problem by assisting with faster decision-making regarding patient admissions; yet many studies neglect to focus on the clinical relevance and practical applications of these AI solutions.

**Objective:**

This study aimed to evaluate the clinical relevance of an AI model in predicting patient admission from the ED to hospital wards and its potential impact on reducing the time needed to make an admission decision.

**Methods:**

A retrospective study was conducted using anonymized patient data from St. Antonius Hospital, the Netherlands, from January 2018 to September 2023. An Extreme Gradient Boosting AI model was developed and tested on these data of 154,347 visits to predict admission decisions. The model was evaluated using data segmented into 10-minute intervals, which reflected real-world applicability. The primary outcome measured was the reduction in the decision-making time between the AI model and the admission decision made by the clinician. Secondary outcomes analyzed the performance of the model across various subgroups, including the age of the patient, medical specialty, classification category, and time of day.

**Results:**

The AI model demonstrated a precision of 0.78 and a recall of 0.73, with a median time saving of 111 (IQR 59-169) minutes for true positive predicted patients. Subgroup analysis revealed that older patients and certain specialties such as pulmonology benefited the most from the AI model, with time savings of up to 90 minutes per patient.

**Conclusions:**

The AI model shows significant potential to reduce the time to admission decisions, alleviate ED overcrowding, and improve patient care. The model offers the advantage of always providing weighted advice on admission, even when the ED is under pressure. Future prospective studies are needed to assess the impact in the real world and further enhance the performance of the model in diverse hospital settings.

## Introduction

### Background

Emergency department (ED) crowding is a growing problem that can lead to the deterioration of the quality of health care. This concern is associated with a rise in medical errors made by clinicians [1,2], prolonged patient stay [3], morbidity [4,5], and increased mortality rates [6-8]. In some cases in the Netherlands, standards of health care were not met, resulting in the temporary closure of EDs [9]. The issue of overcrowding is expected to become even more evident in the coming years due to increased life expectancy and increased demand for complex care [10,11].

In recent years, studies have been extensively exploring the issue of ED crowding [12-14]. In a conceptual model, ED crowding is divided into three interdependent components: (1) the input component, (2) the throughput component, and (3) the output component [15]. Changes in one of these components can contribute to the ED length of stay and therefore ED crowding [16]. Each component comprises multiple factors that can influence the overcrowding problem both independently and through interaction with factors within or outside the component [13]. Focusing specifically on the throughput component, key factors have been identified, namely the experience level of staff [17], shortages of staff within the ED [18,19], availability of beds in the ED [20], delays in test results, and disposition decisions [21].

To address the problem of overcrowding, solutions can be pursued both within the ED and through broader changes at the hospital. Within the ED, particularly concerning the throughput component, studies have investigated, for example, the implementation of fast-track systems [22,23], adjustments in triage models [24], and the rising application of artificial intelligence (AI) solutions [25].

Currently, several studies are investigating the potential of AI solutions to mitigate the overcrowding problem in the ED. Some promising results have been reported in areas of patient admission to inpatient units and intensive care units or discharge from the ED, thereby impacting the duration of stay in the ED [21]. However, these AI models can vary significantly in their functioning, often using diverse parameters at various time points during ED admission [26-32]. While these studies tend to focus on the technical performance of the models, they often neglect to consider their practical relevance and applicability within health care settings [33-35].

In this study, an AI model was developed for decision support in the ED. Moreover, the retrospective model predictions were evaluated with updates occurring every 10 minutes based on the most current patient data. This AI model showed the health care professionals and residents the probability of admission to a hospital ward from the ED.

### Aim

The clinical relevance of the AI decision support system was evaluated by analyzing the decision-making time. This evaluation involves retrospectively examining whether the model can reduce the time required for an admission decision, thereby potentially decreasing ED length of stay and alleviating the overcrowding problem.

## Methods

### Study Design

In this retrospective study, anonymized patient records from the Dutch St. Antonius Hospital were collected from January 2018 to September 2023. The St. Antonius Hospital in the Netherlands has 2 different locations where emergency care is provided, and it is a level 2 trauma center in an urban setting. A total of 41,000 patients present to the EDs each year at the St. Antonius Hospital. The data up until May 2022 were earmarked for the development and assessment of our AI model.

As the emphasis of this study was on the clinical impact rather than the complexities of model development and evaluation, detailed information regarding the development of the model is provided in Multimedia Appendix 1, which includes a comprehensive overview of the preprocessing steps, feature selection, and models considered during the development phase. The development of the model was based on the study by De Hond et al [32]. Admissions in this study comprised patients treated in the EDs of St. Antonius Hospital. Patients who explicitly declined to provide consent for the use of their data in any research context were excluded. Additional exclusion criteria encompassed patients younger than 18 years.

The AI model predicts the admission probability as a percentage. If this percentage exceeds 50%, the model classifies the case as “admission.” Once the threshold is reached, the decision is final and cannot be reversed. This design choice was made to mimic clinical commitment, thereby reflecting a realistic clinical decision-making context in which a patient admission decision is typically irreversible once made.

### Data Collection

Features were extracted from the dataset by performing several steps during the data cleaning and transforming phase. A detailed list of these features, along with a comprehensive explanation of the data-cleaning process, is provided in Table S1 in Multimedia Appendix 1. For model development, the dataset collected between January 1, 2018, and May 15, 2022, was split in an 80:20 ratio randomly; 80% (105,000/131,250) of the data was used for training the AI model, and 20% (26,250/131,250) was used for testing.

To evaluate the AI model’s performance in predicting ED admissions, a dataset including all ED visits from May 16, 2022, to September 1, 2023, was created. This dataset was designed to mimic real-world scenarios, allowing us to assess the model’s clinical performance in a controlled retrospective setting. This dataset was referred to as the evaluation dataset. Admission data were divided into 10-minute intervals, starting from the initial 0 minutes up to 3 hours. This segmentation reflected clinical decision-making by providing the model with the most recent information available at each time point. By checking new predictions every 10 minutes, we could analyze how changes in these predictions supported clinical decision of admission to the ward. This method tested the model’s ability to make accurate predictions with limited and progressively updated information, reflecting typical emergency settings.

Thus, 3 datasets were used: 1 for training the model, 1 for testing during the development phase, and 1 evaluation dataset to calculate the time saved by the AI model compared to admission decisions made by ED health care professionals.

### Model Evaluation

#### General Performance

Different analyses were conducted using the test and the evaluation datasets. General performance metrics such as accuracy, precision, and recall, were calculated using the test dataset.

#### Primary Outcome

The primary outcome measure was the difference in decision-making time between the AI model and the admission decision made by the clinician. The decision of the clinician was defined as the duration from a patient’s arrival at the ED to the time an admission order was placed or when the patient was informed by a health care professional that admission was not required, and discharge was appropriate. This outcome was evaluated against the AI model’s prediction when the predicted admission probability exceeded 50%. When the probability of admission exceeded the threshold, the model predicted an admission and could not revert to “discharge from ED” prediction. This influenced the metrics over time. The health care professional’s admission order served as the benchmark for this comparison. This analysis was performed using the evaluation dataset.

#### Secondary Outcome

The secondary outcome focused on the full patient group and true positive patients predicted by the AI model across various subcategories and baseline calculations. The patients correctly assessed by the model as admitted were classified as the true positive category. These baseline calculations represented the majority class assumption. These subcategories included age groups, medical specialties, triage categories, and different parts of the day. These results were obtained using the evaluation dataset, including the constraint that once the model predicts admission, it cannot be revised to discharge. Each subcategory was analyzed to determine its impact on admission decision time. These categories were also cross analyzed to assess coherence. These categories were chosen based on the assessment of importance by an ED clinician.

The ages of patients upon their arrival at the ED were analyzed. Patient ages were recorded and categorized into 10-year intervals such as 18 to 27, 28 to 37, and other age groups.

The medical specialty assigned at the time of ED arrival was examined, as different specialties could lead to varied outcomes. In this study, specialties included the top 10 presented specialties in the ED.

Upon arrival, patients were assigned a triage category indicating the urgency of their condition. The triage categories in the Netherlands range from U0 to U5, with U0 being the highest critical state of health.

The data were analyzed based on the arrival time of day, which may influence outcomes due to varying compositions of staff present in the ED. The data were segmented into 4 periods: night (midnight to 6 AM), morning (6 AM to noon), afternoon (noon to 6 PM), and evening (6 PM to midnight).

Baseline calculations were also performed for each subcategory to provide a reference point. This baseline represented the scenario in which all cases were assumed to result in admission (ie, if most patients are admitted, then a “yes, admit this patient” prediction for all patients is made). For each subcategory, the percentage of correct predictions under this baseline assumption was calculated and compared against the AI model’s performance. This showed us how the current situation could potentially be adapted and improved.

In addition, the feature importance of the model was examined to assess the impact of individual features on the model’s inclusion decision. A higher value indicated a greater contribution to the final decision of the model. These findings can inform the selection of features for future models and support clinical decision-making.

### Ethical Considerations

This study was reviewed and approved by the St. Antonius Hospital’s local review committee (approval R&D/Z24.050). No informed consent from patients was required for this study, as it did not involve any additional risks or burdens for patients. Patients whose anonymized data were used for this study received no compensation. All patient data were processed anonymously and stored on a secure server with restricted access, in accordance with data protection laws and regulations.

## Results

### General Performance

The AI support model for decision prediction in the ED was an Extreme Gradient Boosting with an accuracy of 0.81, precision of 0.78, recall of 0.73, *F*_1_-score of 0.75, and a receiver operating characteristic area under the curve of 0.89 on the test dataset. The final hyperparameters used for this model included a colsample_bytree of 0.7, γ of 0.0, learning rate of 0.1, max_depth of 15, and min_child_weight of 7.

### Primary Outcome

The median durations of admission order placement by health care professionals were compared to the time required by the AI model on retrospective data to make equivalent decisions. Health care professionals required a median time of 151 (IQR 95-228) minutes to make admission decisions, decreasing to 131 (IQR 75-201) minutes for the admitted patient population. In contrast, the AI model achieved a median decision time of 20 (IQR 0-40) minutes for the correct prediction. This represented a time saving of 111 minutes per patient for correct predictions when using the AI model.

**Figure 1:**
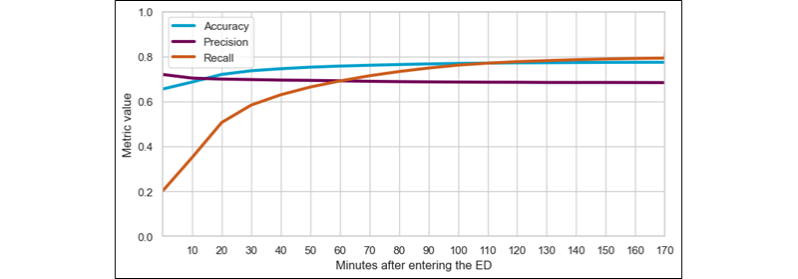
Evolution of the performance metrics during the stay of the patient in the emergency department (ED).

Figure 1 shows the performance metrics of the AI model. It illustrates that the quality of the admission predictions evolves over time. Initially, the model failed to capture many cases, but it eventually achieved a precision of 80% (24,696/30,870). Nevertheless, it still generated 30% (9261/30,870) to 35% (10,805/30,870) false positives, which may lead to excessive and unnecessary alarms for the medical staff. These metrics are slightly different from the general performance, since a stricter admission rule was applied; once the model admits a patient, this decision cannot be reversed. These results also showed that, later during the ED stay, the AI model made fewer mistakes than earlier.

### Secondary Outcomes

#### Age

**Figure 2:**
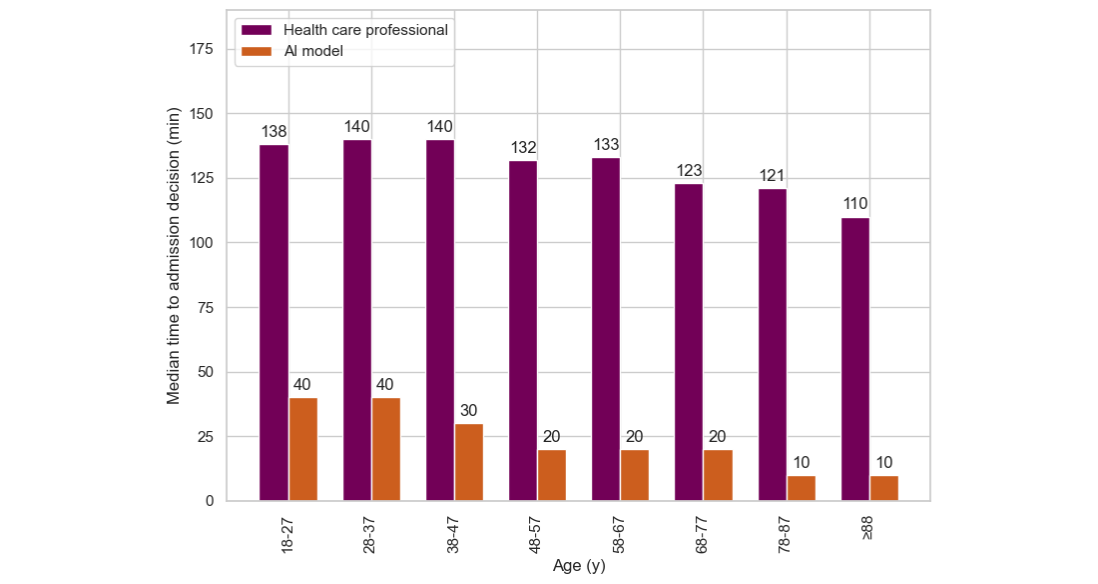
Time to admission decision for all true positive predicted patients, stratified by age group. The greater the difference, the greater the potential time saving. AI: artificial intelligence.

Figure 2 and Table 1 show that younger patients (aged 18-27 y) had a median current time of 137 minutes in the ED, with a substantial improvement in time saved per patient (100 min). The precision of 0.51 and the recall of 0.46 suggested that younger patients were often misclassified. In contrast, the older adult population (aged 78-87 y and ≥88 y) presented the greatest clinical impact. Their admission times (for the true predicted patients) were reduced by 120 and 110 minutes per patient, respectively, and the model achieved a strong precision of 0.75 and 0.78 and a recall of 0.90 and 0.91.

For the 18- to 27-year age group, the model’s accuracy (84%) matched the baseline of assuming no admissions (84%). For all other age groups, the model consistently outperformed the baseline.

**Table 1:** Model performance for different age groups, showing clear differences between younger and older patients.

Age group (y)	True positives, n (%)	True negatives, n (%)	False positives, n (%)	False negatives, n (%)	Precision	Recall	Accuracy	Majority accuracy
18-27 (n=4658)	346 (7.4)	3561 (76.4)	338 (7.3)	413 (8.9)	0.51	0.46	0.84	0.84
28-37 (n=5312)	536 (10.1)	3689 (69.4)	452 (8.5)	635 (12)	0.54	0.46	0.80	0.78
38-47 (n=4730)	675 (14.3)	3058 (64.7)	531 (11.2)	466 (9.9)	0.56	0.59	0.79	0.76
48-57 (n=6064)	1418 (23.4)	3153 (52)	903 (14.9)	590 (9.7)	0.61	0.71	0.75	0.67
58-67 (n=7330)	2484 (33.9)	2991 (40.8)	1297 (17.7)	558 (7.6)	0.66	0.82	0.75	0.58
68-77 (n=9437)	4257 (45.1)	2848 (30.2)	1713 (18.2)	619 (6.6)	0.71	0.87	0.75	0.52
78-87 (n=7444)	3806 (51.1)	1951 (26.2)	1265 (17)	422 (5.7)	0.75	0.90	0.77	0.57
≥88 (n=2332)	1265 (54.2)	590 (25.3)	349 (15)	128 (5.5)	0.78	0.91	0.80	0.60

#### Medical Specialty

**Figure 3:**
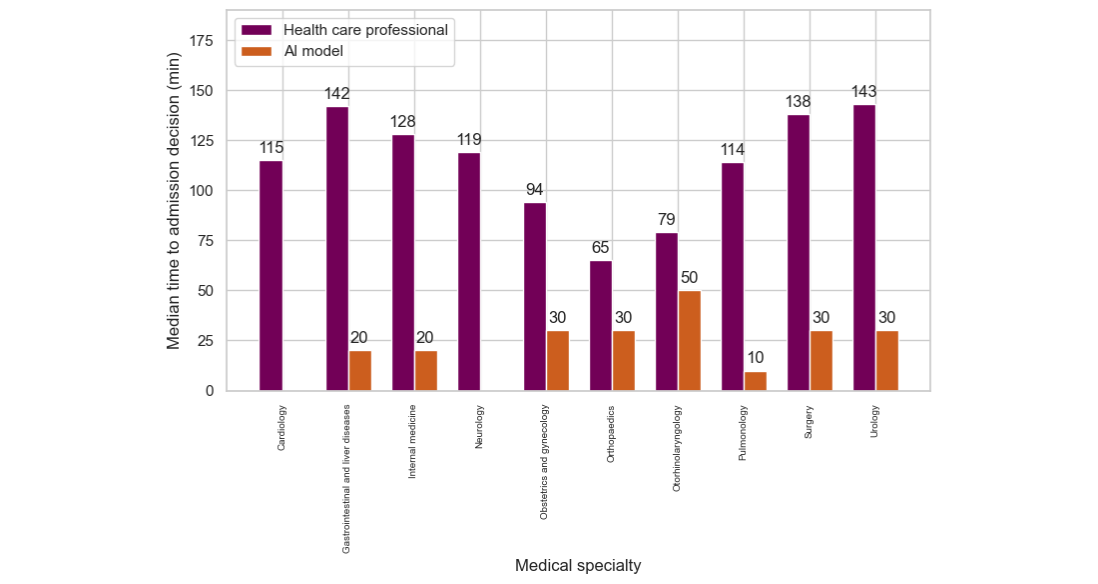
Time to admission decision for all true positive predicted patients, stratified by medical specialty. The greater the difference, the greater the potential time saving. AI: artificial intelligence.

Table 2 shows that pulmonology and gastrointestinal and liver disease cases showed a recall rate of higher than 0.90 and a precision value of more than 0.7. With this balance between recall and precision, the model ensured that the clinical risks of missed admissions (false negatives) were minimized, while the clinical impact of unnecessary admissions (false positives) on hospital capacity remained manageable. By contrast, specialties such as neurology, surgery, otorhinolaryngology, and internal medicine demonstrated greater challenges. For example, neurology had a recall of 0.82 but a lower precision of 0.52, with a significant number of false positives (1438/4659, 30.9%). This resource burden reflected the difficulty in assessing neurological symptoms. Figure 3 shows that across all specialties, the model consistently outperformed the baseline accuracy. It also shows that in neurology and cardiology, the admission decision time was 0 minutes for the AI model.

**Table 2:** Model performance for different medical specialties.

Medical specialty	True positives, n (%)	True negatives, n (%)	False positives, n (%)	False negatives, n (%)	Precision	Recall	Accuracy	Majority accuracy
Obstetrics and gynecology (n=1003)	126 (12.6)	648 (64.6)	65 (6.5)	164 (16.4)	0.66	0.43	0.77	0.71
Urology (n=1931)	658 (34.1)	791 (41)	247 (12.8)	235 (12.2)	0.73	0.74	0.75	0.54
Orthopedics (n=1878)	242 (12.9)	1411 (75.1)	127 (6.8)	98 (5.2)	0.66	0.71	0.88	0.82
Neurology (n=4659)	1543 (33.1)	1338 (28.7)	1438 (30.9)	340 (7.3)	0.52	0.82	0.62	0.60
Gastrointestinal and liver diseases (n=2502)	1436 (57.4)	321 (12.8)	618 (24.7)	127 (5.1)	0.70	0.92	0.70	0.70
Pulmonology (n=6068)	3170 (52.2)	1519 (25)	1034 (17)	345 (5.7)	0.75	0.90	0.77	0.77
Otorhinolaryngology (n=643)	38 (5.9)	512 (79.6)	26 (4)	67 (10.4)	0.59	0.36	0.86	0.84
Internal medicine (n=7810)	3598 (46.1)	2342 (30)	1399 (17.9)	471 (6)	0.72	0.88	0.76	0.52
Surgery (n=17,720)	2852 (16.1)	11,758 (66.4)	1290 (7.3)	1820 (10.3)	0.69	0.61	0.82	0.74
Cardiology (n=916)	471 (51.4)	154 (16.8)	265 (28.9)	26 (2.8)	0.64	0.95	0.68	0.54

#### Triage Categories

**Figure 4:**
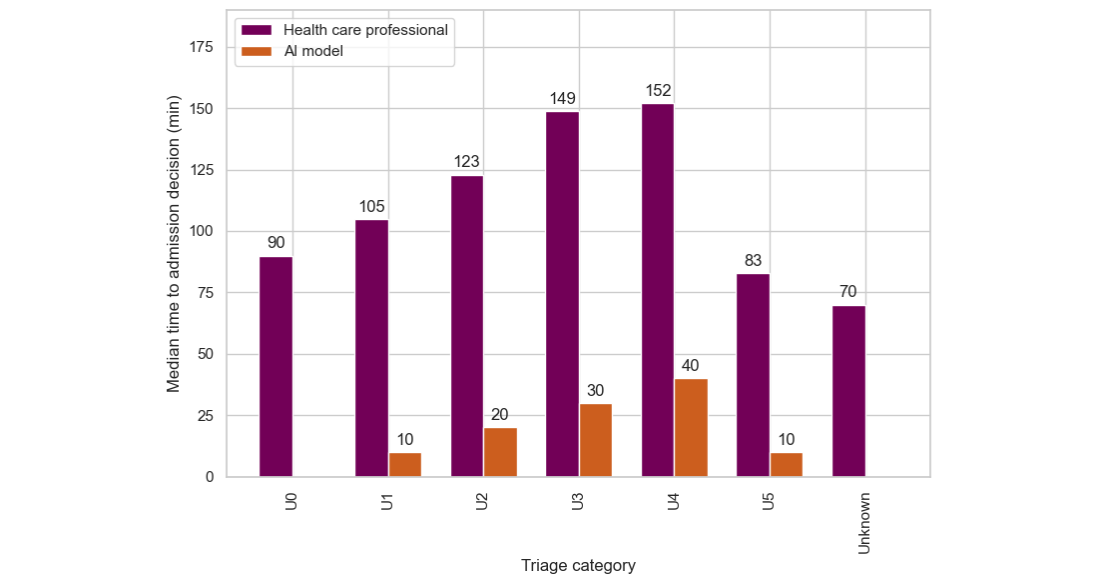
Time to admission decision for all true positive predicted patients, stratified by urgency level (triage category). The greater the difference, the greater the potential time saving. AI: artificial intelligence.

Figure 4 and Table 3 show that the model performs particularly well for the most critical patients (U0 and U1 categories), where the precision and recall ensure that almost all high-risk admissions are caught in time, saving crucial minutes. The system saves 80 minutes for the U0 category and 100 minutes for the U1 category per true positive predicted patient. However, challenges emerged in the U3 and U4 categories, where lower precision and recall indicated a notable number of false positives and false negatives. In such cases, unnecessary admissions could burden resources, while missed cases could endanger lives, indicating that improvements in midtier urgency cases could significantly impact ED efficiency. The baseline slightly outperformed the model for the U0 category, whereas the model excelled in all other triage categories. Figure 4 shows that the admission decision time was 0 minutes for the U0 and unknown categories when using the AI model.

**Table 3:** Model performance across triage urgency levels.

Triage category	True positives, n (%)	True negatives, n (%)	False positives, n (%)	False negatives, n (%)	Precision	Recall	Accuracy	Majority accuracy
Unknown (n=1355)	322 (23.8)	865 (63.8)	93 (6.9)	75 (5.5)	0.78	0.81	0.88	0.71
U0 (n=178)	170 (95.5)	0 (0)	6 (3.4)	2 (1.1)	0.97	0.99	0.96	0.97
U1 (n=4257)	2442 (57.4)	529 (12.4)	1172 (27.5)	114 (2.7)	0.68	0.96	0.70	0.60
U2 (n=18,403)	8077 (43.9)	5670 (30.8)	3253 (17.7)	1403 (7.6)	0.71	0.85	0.75	0.52
U3 (n=17,362)	3170 (18.3)	10,731 (61.8)	1829 (10.5)	1632 (9.4)	0.63	0.66	0.80	0.72
U4 (n=5733)	603 (10.5)	4032 (70.3)	494 (8.6)	604 (10.5)	0.55	0.50	0.81	0.79
U5 (n=19)	3 (15.8)	14 (73.7)	1 (5.3)	1 (5.3)	0.75	0.75	0.89	0.79

#### Part of the Day

**Figure 5:**
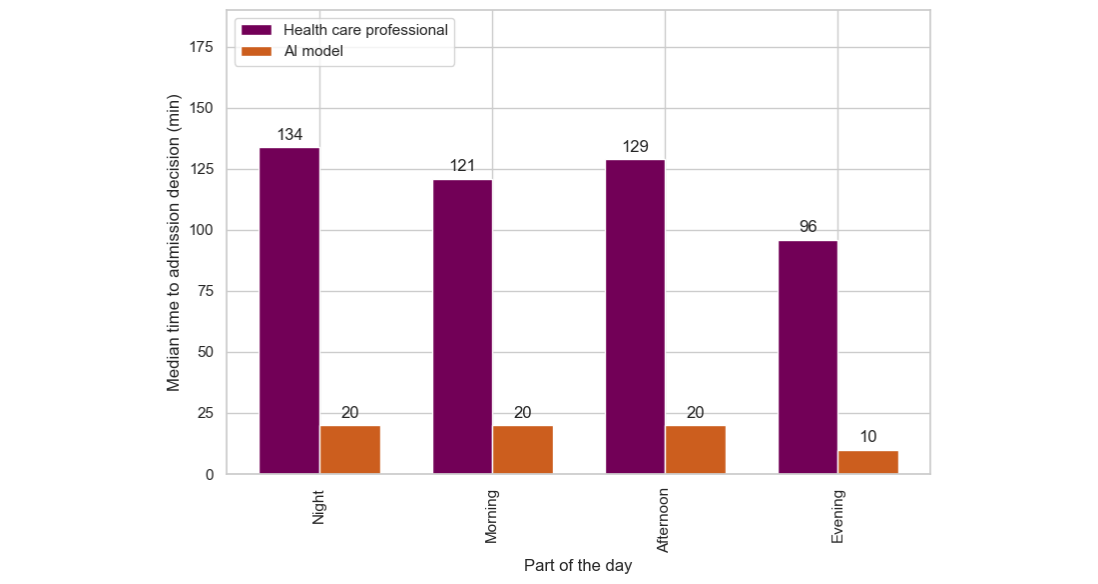
Time to admission decision for all true positive predicted patients, stratified by the part of the day a patient entered the emergency department. The greater the difference, the greater the potential time saving. AI: artificial intelligence.

Table 4 shows that performance varied depending on the time of day. Between noon and 6 PM, when the ED experienced its highest patient volume, the model achieved its best overall performance. A recall of 0.79 indicated that most patients needing admission were accurately flagged, saving, on average, 100 minutes per (true positive predicted) patient. Conversely, during quieter night shifts (midnight to 6 AM), the model’s precision decreased to 0.67. However, it exhibited a higher recall rate compared to the afternoon shift. Figure 5 shows that across all time periods, the model outperformed the baseline.

**Table 4:** Model performance across arrival time periods, showing consistency in performance.

Arrival time	True positives, n (%)	True negatives, n (%)	False positives, n (%)	False negatives, n (%)	Precision	Recall	Accuracy	Majority accuracy
Night (midnight-6 AM; n=3311)	1300 (39.3)	1175 (35.5)	637 (19.2)	199 (6)	0.67	0.87	0.75	0.55
Morning (6 AM-noon; n=11,251)	3259 (29)	5280 (46.9)	1637 (14.5)	1075 (9.6)	0.67	0.75	0.76	0.61
Afternoon (noon-6 PM; n=22,093)	6696 (30.3)	10,582 (47.9)	3061 (13.9)	1754 (7.9)	0.69	0.79	0.78	0.62
Evening (6 PM-midnight; n=10,652)	3532 (33.2)	4804 (45.1)	1513 (14.2)	803 (7.5)	0.70	0.81	0.78	0.59

#### Feature Importance

The results of the feature importance are shown in Multimedia Appendix 2. Table S1 in Multimedia Appendix 2 shows the top 20 most influential features, demonstrating that orders for inflammation, orders for kidney function, orders for blood count, and orders for blood cultures had the strongest influence.

#### Subcategory Coherence

**Figure 6:**
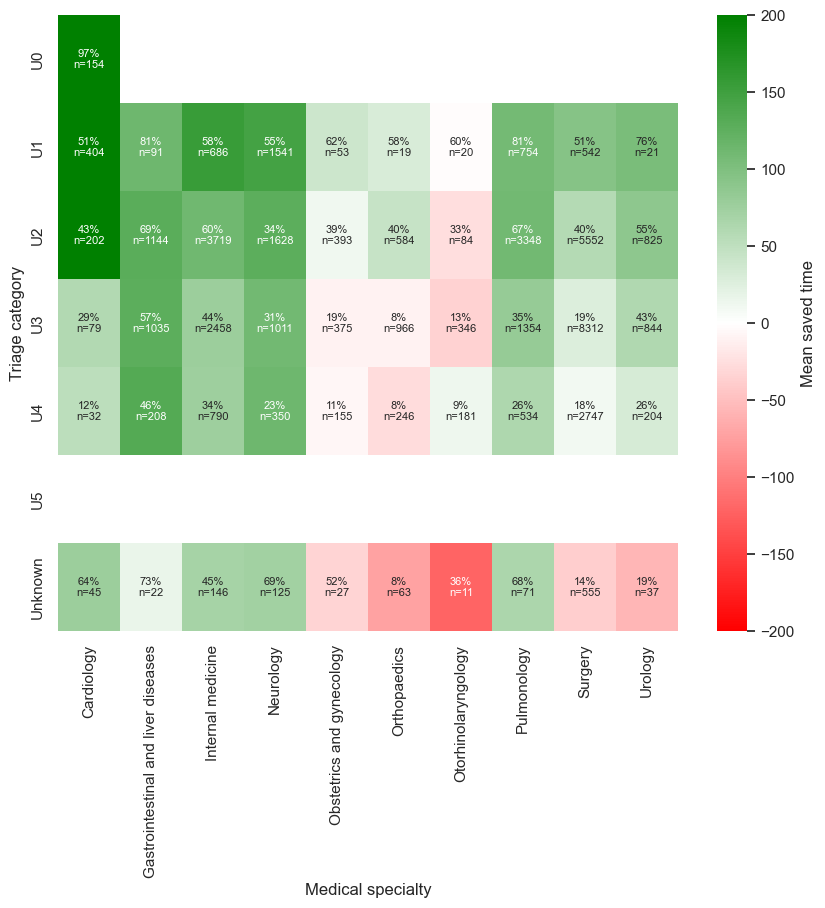
Saved time per patient between the medical specialty and triage category.

**Figure 7:**
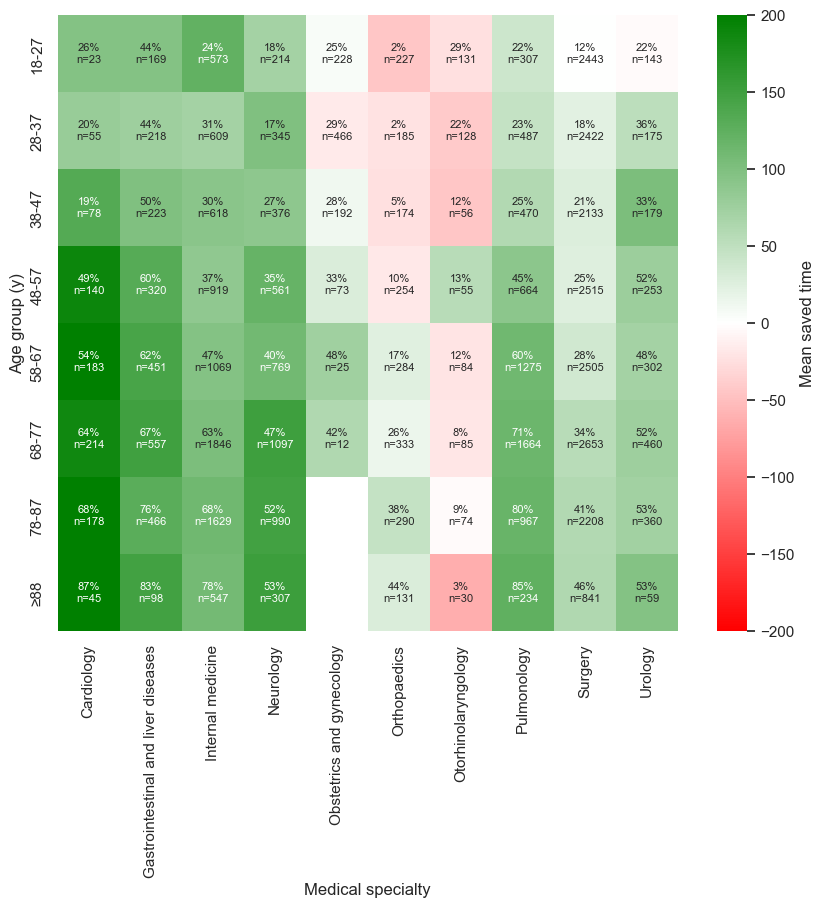
Saved time per patient between medical specialty and age group.

**Figure 8:**
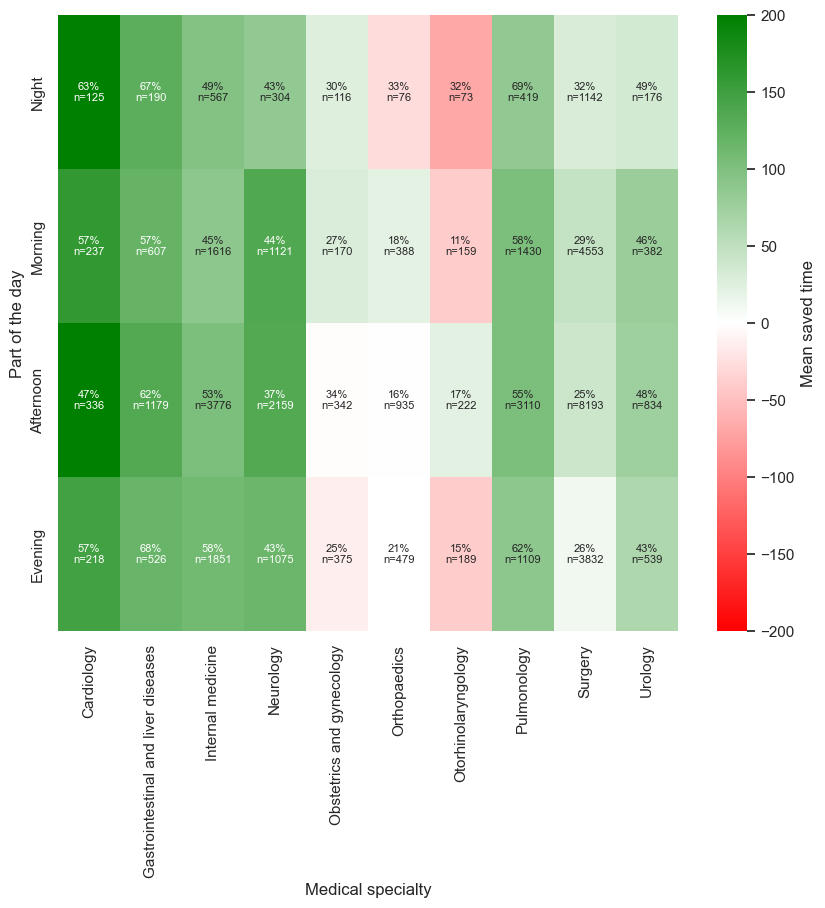
Saved time per patient between the medical specialty and part of the day.

**Figure 9:**
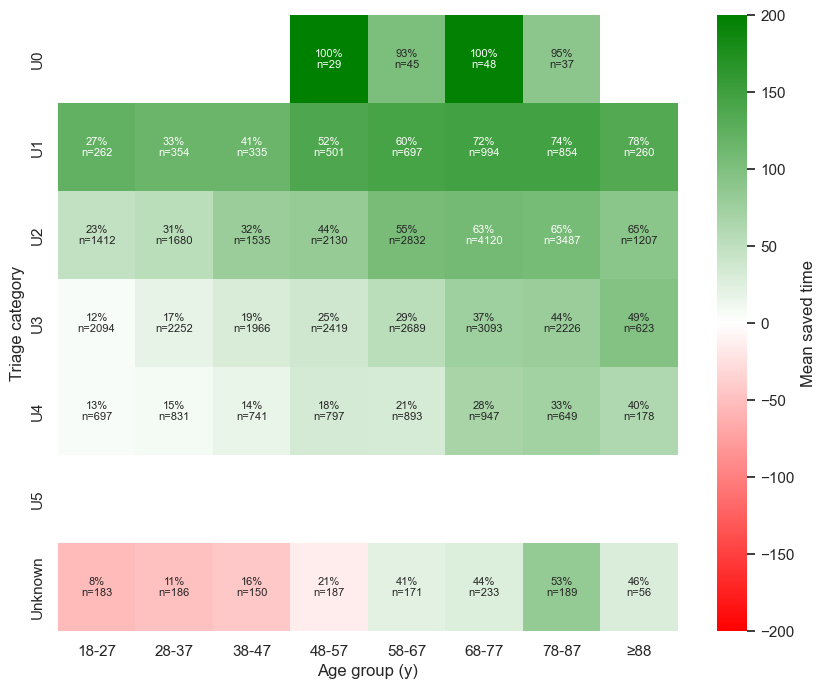
Saved time per patient between triage category and age group.

**Figure 10:**
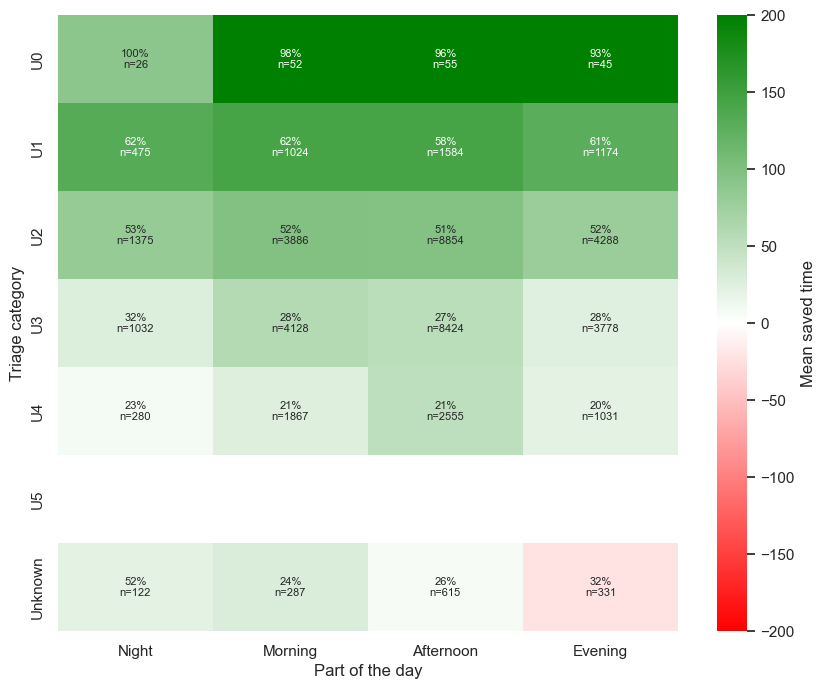
Saved time per patient between the triage category and the part of the day.

**Figure 11:**
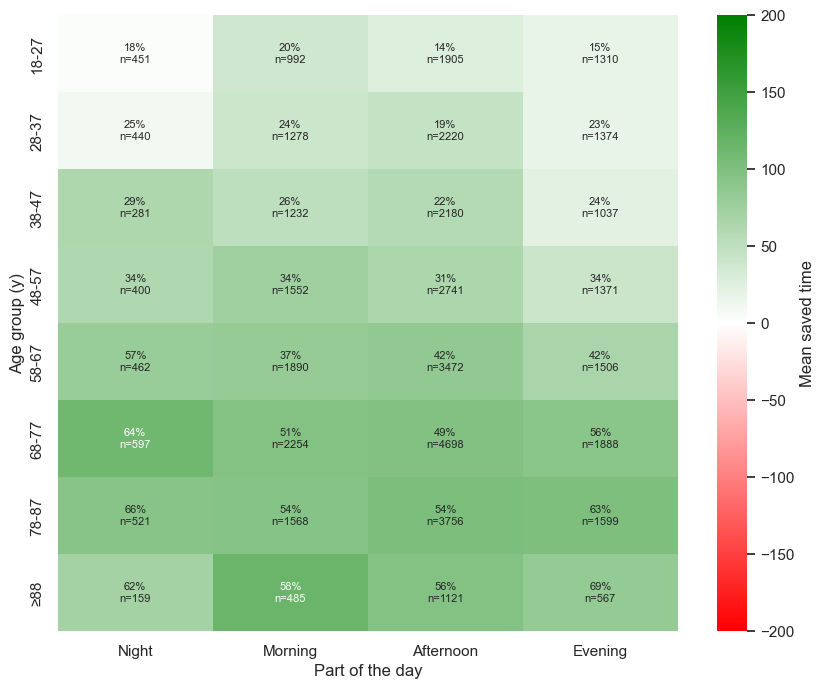
Saved time per patient between the age groups and part of the day.

Further analysis was conducted to evaluate the coherence of subcategories within the datasets. This additional layer of analysis aimed to ensure consistency in the results and provided a deeper understanding of the underlying patterns (Figures 6-11). This is presented in Multimedia Appendix 3.

## Discussion

### Principal Findings

Our study addresses the potential for integrating an AI decision model into clinical practice by not only developing an AI model using Extreme Gradient Boosting but also evaluating its clinical relevance through a 10-minute evaluation dataset. Many models rely on static inputs and technical performance, without addressing integration into clinical workflows [26-32]. Unlike previous studies, our model revises its admission decision at 10-minute intervals, enhancing clinical relevance and facilitating seamless integration into the clinical workflow.

It demonstrated that using AI to support the physicians in the ED has the potential to reduce time to an admission decision by 111 (IQR 59-169) minutes per correctly predicted patient, thereby improving the quality of care and reducing pressure on hospital resources. The model achieves an accuracy of 0.81, an *F*_1_-score of 0.75, and a receiver operating characteristic area under the curve of 0.89. Nevertheless, these findings should be interpreted with caution, as both clinical relevance and performance are likely to be lower when implemented in an actual clinical workflow compared to existing literature [28,32]. Unlike previous work, our approach integrates iterative data updates every 10 minutes and checks the clinical impact of AI decision-making. This study provides a practically oriented contribution by demonstrating how AI can support timely decision-making, especially for less experienced clinicians.

Importantly, the model does not negatively impact patient safety. In the case of a true positive prediction, the patient is transferred to the correct department quickly and receives more specific treatment faster. In the case of a false negative prediction, the patient is transferred to the department as quickly as they would have without the AI model. In the case of a false positive prediction, it only leads to additional work for the employees. However, in this hospital’s case, this did not lead to clinically significant consequences.

### Limitations

The findings suggest that AI models can be effectively used to enhance the decision-making processes in the ED, leading to reduced time to admission decisions and potentially improving patient outcomes.

One data limitation of this study is that the model does not include radiological image results, blood gas, and free-text clinical notes, all of which are critical for a comprehensive patient assessment. Incorporating these data types could improve the model’s metrics and reliability. Additionally, the model does not account for data from previous appointments, which could provide valuable context and insights into a patient’s history and potential risks.

Another notable consideration is the potential consequence of faster admission decisions from the ED, namely a false admission prediction. This could lead to an unnecessary order being sent to the urgency coordinator to arrange a bed that is ultimately not required, resulting in wasted time for staff and disrupting operational workflows. In consultation with the urgency coordinators, it was agreed that this is a consequence that has minimal impact on the St Antonius Hospital.

### Considerations (of Implications) for Implementation

While the AI model shows promise in an ideal scenario, real-world implementation will still face several challenges. Even with AI recommendations, delays in placing orders are likely to occur due to factors such as health care professionals being occupied or requiring additional time to assess patients. Additionally, factors such as the department’s workload, patient flow, and the need for patient transfers within the region can further affect the time to admission decision. In practice, the time difference may not be as significant as predicted by the model alone. A combination of health care professionals and AI models will need to work together, and this interaction should be explored in a prospective study, which is planned [35]. This combination of AI and health care professionals is also what could improve the false positive rate and thus the model, compared to just working with the AI model.

### Future Directions

A prospective study is recommended to evaluate the actual impact of the model on ED length of stay in a real-world setting. To improve predictive accuracy, such a study should assess the model's performance in the clinical setting. In addition, incorporating additional data sources such as imaging results and patient history, could enhance the model’s applicability in a real-world setting.

### Conclusions

ED overcrowding poses a challenge to health care systems, contributing to delays in treatment, increased medical errors, and compromised patient outcomes. This study was motivated by the urgent need to expedite the decision-making process within the ED to reduce patient ED length of stay.

To address this, we developed and evaluated an AI-based decision support model capable of predicting hospital admissions from the ED. Unlike previous studies that primarily focused on technical model performance, our work emphasizes clinical relevance through real-time decision-making via 10-minute interval updates, mimicking the dynamics of actual ED workflows.

The model achieved a precision of 0.78 and a recall of 0.73. In a retrospective dataset, the AI model was able to reduce the median time to admission order by 111 (IQR 59-169) minutes for correctly predicted admissions, potentially alleviating ED overcrowding and improving patient care. In addition, it offers the advantage of consistently providing weighted advice on admission, even when the ED is under pressure.

These findings demonstrate that integrating AI decision support into clinical workflows has the potential to speed up decisions, reduce ED overcrowding, and thus improve patient care. Future prospective studies are essential to validate these results in real-world settings.

## Appendix

Multimedia Appendix 1 Model development.

Multimedia Appendix 2 Feature importance.

Multimedia Appendix 3 Subcategory coherence.

Multimedia Appendix 4 CREMLS (Consolidated Reporting Guidelines for Prognostic and Diagnostic Machine Learning Models) checklist.

## Abbreviations

AI: artificial intelligence
ED: emergency department

## Data Availability

The datasets generated or analyzed during this study are available from the corresponding author on reasonable request.
