# Supplementary material for: Evaluating an AI Decision Support System for the Emergency Department: Retrospective Study
Source: JMIR AI. 2026 Jan 26;5:e80448. doi: 10.2196/80448 (PMC12887564; doi:10.2196/80448)
Supplement: Multimedia Appendix 1 [file ai_v5i1e80448_app1.docx]

### Multimedia appendix 1 - Model development

The design of the AI prediction model for admission descision is described in this Chapter. All chapters represent steps within the model development using python 3.9.

#### Data extraction

The anonymised medical records of all patients presented between January 2018 and September 2023 at the emergency departments of the two separate locations of the St. Antonius Hospital in the Netherlands were used for the design, validation and real world impact of an AI model. The St. Antonius hospital is a level-II trauma center in an urban setting.

#### Data cleaning

All patients who were presented to one of the EDs (Nieuwegein or Utrecht) of the St. Antonius Hospital were eligible for inclusion. All patients 18 years of age or older were included. Patients were excluded if age was over 110 or gender was unknown.

Several steps were taken as part of the data-cleaning phase. Blood pressure was separated into upper and lower pressure, the temperature was converted from Fahrenheit to Celsius, and all orders in which the order time was not filled were removed, as it cannot be determined whether the order was placed during the ED stay. For the categorical features specialty, primary complaint, arrival method, referral type, location, gender, and arrival time, one-hot encoding is used.

We also looked at missing values, normal values, lower limit, and upper limit. In some cases, there might be odd lab values among them due to a typo, for instance. Together with the specialist, it is discussed which normal value, lower limit, and upper limit to use. Anything not within the range (of lower to upper limit) or missing is replaced by the normal value. See Table S1 for more information. For pulse rate, the normal value is based on multiple parameters. This can be seen in [1].

Table S1, Imputed values

| **Components** | **Lower limit** | **Upper limit** | **Imputed normal value** |
| --- | --- | --- | --- |
| SpO2 | 0 | 100 | 98 |
| Pulse rate | 0 | 300 | Ostchega et al. [1] |
| Overpressure | 0 | 250 | 120 |
| Underpressure | 0 | 150 | 80 |
| Respiratory rate | 0 | 60 | 12 |
| Temperature | 20 | 43 | 37 |
| Pain score | 0 | 10 | 0 |
| Glasgow Coma Scale | 3 | 15 | 15 |
| Platelets | 0 | 3000 | 250 |
| MCV | 35 | 150 | 98 |
| Hematocrit | 0,1 | 0,8 | 0,4 |
| CRP | 0 | 800 | 3 |
| gamma-GT | 0 | 6000 | 30 |
| LDH | 0 | 20.000 | 175 |
| Urea | 0 | 200 | 3 |
| Hemoglobin | 0,5 | 20 | 8 |
| Potassium | 1 | 15 | 4 |
| Calcium | 0,1 | 8 | 2,2 |
| CK | 1 | 200.000 | 250 |
| Creatinine | 0 | 2000 | 70 |
| Alk. phosphatase | 0 | 5000 | 80 |
| ASAT | 0 | 15.000 | 20 |
| ALAT | 0 | 8000 | 20 |
| Leukocytes | 0 | 600 | 5 |
| Sodium | 100 | 180 | 140 |
| Segments | 0 | 20 | 2 |

#### Target and feature selection

Admission was defined as being admitted to any inpatient ward within the hospital, or admitted to any other hospital. Also, patients who passed away during the ED visit are defined as being admitted. All others are considered “discharged”.

Feature selection is based on the study by De Hond et al [2]. Some features with a high correlation are removed; these are mean atrial pressure and hematocrit. Also, based on the process analysis, some laborers are categorized. So, is an inflammation a CRP lab order? A Blood sample order is for hemoglobin, leukocytes, platelets, and MCV. Electrolyte/kidney function is for sodium, potassium, creatinine, and urea. Liver function, these are the values ALAT, ASAT, and alk. Phosphatase. And as the last two, Muscle function with LD and CK, and calcium with just calcium. This can also be seen in the last two columns of Table S2.

Table S2, Final features grouped by information type

| **Patient** | **Triage information** | **Vital signs** | **Orders** | **Lab order** | **Lab results** |
| --- | --- | --- | --- | --- | --- |
| Gender | Urgency category | SpO2 | Orders Electrocardiogram | Inflamation | CRP |
| Age | Primary complaint | Overpressure | Bucky | Blood sample | Hemoglobin |
|  | Specialty | Underpressure | CT |  | Leukocytes |
|  | Method of arrival | Pulse rate | Dexa |  | Platelets |
|  | Referral type | Respiratory rate | Echo |  | MCV |
|  | Location (NG/UTR) | Temperature | MRI | Electrolyte/kidney function | Sodium |
|  | Daypart | Pain score | Blood values |  | Potassium |
|  | Weekday | Glasgow Coma Scale | Consultations |  | Kreatinine |
|  |  | SpO2 - measured yes/no |  |  | Urea |
|  |  | Peak pressure - measured yes/no |  | Liver function | ALAT |
|  |  | Diastolic pressure - measured yes/no |  |  | ASAT |
|  |  | Pulse rate - measured yes/no |  |  | Alk. phosphatase |
|  |  | Respiratory rate - measured yes/no. |  |  | gamma-GT |
|  |  | Temperature - measured yes/no |  | Muscle function | LD |
|  |  | Pain score - measured yes/no |  |  | CK |
|  |  | Glasgow Coma Scale - measured yes/no |  | Calcium | Calcium |

#### Train and test set

The data on patients presented between January 1^st,^ 2018, and May 15^th^, 2022, was split with a 5-fold cross-validation. During training, all aforementioned features (Table S2) were used to predict hospital admission of these patients. Validation was done on the rest 20% of the data. The data from May 16^th^, 2022, till the first September 2023, was used for the evaluation dataset. This has been described in Chapter Method, subchapter Data.

#### Model

For training the models, XGBoost [3], Logistic classifier and Random Forest [3] were used as algorithm options. Technical performance of the AI model was assessed by accuracy, precision, recall, balanced F-score (F1), and area under the curve (AUC). For the different models, there are different hyperparameters. The options that were used to develop the model are shown, per model, in Table S3.

Table S3, Hyperparameters

| **Model** | **Parameter** | **Setting option** |
| --- | --- | --- |
| Logistic Regression Classifier | penalty | 'l1', 'l2', 'elasticnet', 'none' |
|  | C | 0.001, 0.01, 0.1, 1.0, 10.0, 100.0, 1000.0 |
|  | solver | 'liblinear', 'lbfgs', 'newton-cg', 'sag', 'saga' |
|  | max_iter | 100, 500 |
| Random Forest Classifier | max_depth | 10, 30, 50, 70 |
|  | max_features | 'log2', 'sqrt' |
|  | min_samples_split | 2, 5, 1 |
|  | min_samples_leaf | 1, 2, 4 |
|  | n_estimators | 400, 800, 1200, 1600 |
| XGBoost Classifier | max_depth | 3, 8, 15 |
|  | colsample_bytree | 0.3, 0.7 |
|  | min_child_weight | 1, 3, 5, 7 |
|  | learning_rate | 0.05, 0.1, 0.3 |
|  | gamma | 0.0, 0.2, 0.4 |

## References

1. Ostchega Y, Porter KS, Hughes J, Charles MPH;, Dillon F, Nwankwo T. Resting Pulse Rate Reference Data for Children, Adolescents, and Adults: United States, 1999-2008. Published online 2011.

2. De Hond A, Raven W, Schinkelshoek L, et al. Machine learning for developing a prediction model of hospital admission of emergency department patients: Hype or hope? *Int J Med Inform*. 2021;152. doi:10.1016/j.ijmedinf.2021.104496

3. Chen T, Guestrin C. XGBoost: A scalable tree boosting system. *Proc ACM SIGKDD Int Conf Knowl Discov Data Min*. 2016;13-17-August-2016:785-794. doi:10.1145/2939672.2939785
