# Supplementary material for: Evaluating an AI Decision Support System for the Emergency Department: Retrospective Study
Source: JMIR AI. 2026 Jan 26;5:e80448. doi: 10.2196/80448 (PMC12887564; doi:10.2196/80448)
Supplement: Multimedia Appendix 2 [file ai_v5i1e80448_app2.docx]

### Multimedia appendix 2 – Feature importance

In this chapter the feature importance of the AI model is presented, highlighting the variables that have the greatest imapct on uptake prediction. A higher feature importance value indicates a stronger contribution to the model’s prediction. Table S1 shows the top 20 most influetial features, demonstrating that Orders Inflammation, Orders Kidney Fucntion, Oders Blood Count and Orders Blood Culteres have the highest influence.

Table S1. Top 20 feature importance with original name and English translation

| **Dutch feature name** | **English translation** | **Importance** |
| --- | --- | --- |
| orders_inflammatie | Orders Inflammation | 0.377268 |
| orders_nierfunctie | Orders Kidney Function | 0.219281 |
| orders_bloedbeeld | Orders Blood Count | 0.093691 |
| orders_blood_cultures | Order Blood Cultures | 0.058071 |
| PrimaryChiefComplaint_TRAUMA EXTREMITEIT | Primary Chief Complaint: Trauma Extremity | 0.008046 |
| HospitalService_X_Cardio Thoracale chirurgie | Cardiac Thoracic Surgery | 0.007321 |
| PrimaryChiefComplaint_THORACALE PIJN | Primary Chief Complaint: Thoracic Pain | 0.007248 |
| Acuitylevel | Acuity Level | 0.005725 |
| HospitalService_X_Maag darm en lever ziekten (Gastro-enterologie) | Gastroenterology | 0.005231 |
| PrimaryChiefComplaint_BEENKLACHTEN | Primary Chief Complaint: Leg Complaints | 0.004877 |
| PrimaryChiefComplaint_WEGRAKING | Primary Chief Complaint: Fainting | 0.004844 |
| HospitalService_X_Chirurgie | Surgery | 0.003978 |
| PrimaryChiefComplaint_NEUROLOGISCHE UITVAL | Primary Chief Complaint: Neurological Deficit | 0.003940 |
| PrimaryChiefComplaint_ALGEHELE MALAISE VOLWASSENE | Primary Chief Complaint: General Malaise in Adult | 0.003898 |
| PrimaryChiefComplaint_*Unspecified | Primary Chief Complaint: Unspecified | 0.003893 |
| PrimaryChiefComplaint_TRAUMA NEK | Primary Chief Complaint: Neck Trauma | 0.003481 |
| crp | C-Reactive Protein | 0.003438 |
| PrimaryChiefComplaint_HOOFDPIJN | Primary Chief Complaint: Headache | 0.003400 |
| ArrivalMethod_Eigen vervoer | Arrival Method: Own Transport | 0.003377 |
| PrimaryChiefComplaint_BRAKEN | Primary Chief Complaint: Vomiting | 0.003365 |
