# Supplementary material for: Evaluating an AI Decision Support System for the Emergency Department: Retrospective Study
Source: JMIR AI. 2026 Jan 26;5:e80448. doi: 10.2196/80448 (PMC12887564; doi:10.2196/80448)
Supplement: Multimedia Appendix 3 [file ai_v5i1e80448_app3.docx]

### Multimedia appendix 3 – Subcategories coherence

This chapter describes the relationship between the subcategories. This is shown through a heatmap that includes the target presence, count, and mean saved time per patient (time difference between the AI model and current situation/healthcare specialists). These results can tell us something about the underlying patterns between the subcategories. All cells that contain fewer than 10 patients are removed from the heatmap due to privacy reasons.

#### Medical specialists vs Triage

Figure S1 demonstrates a strong correlation between U0 and more than half of the medical specialties. U1 shows a notable positive relation with Pulmonology. The greatest time savings are observed in Cardiology, specifically woth triage categorie U0, U1 and U2. A weaker correlations is identified for U5, particularly in relation to a large number of medical specialties.


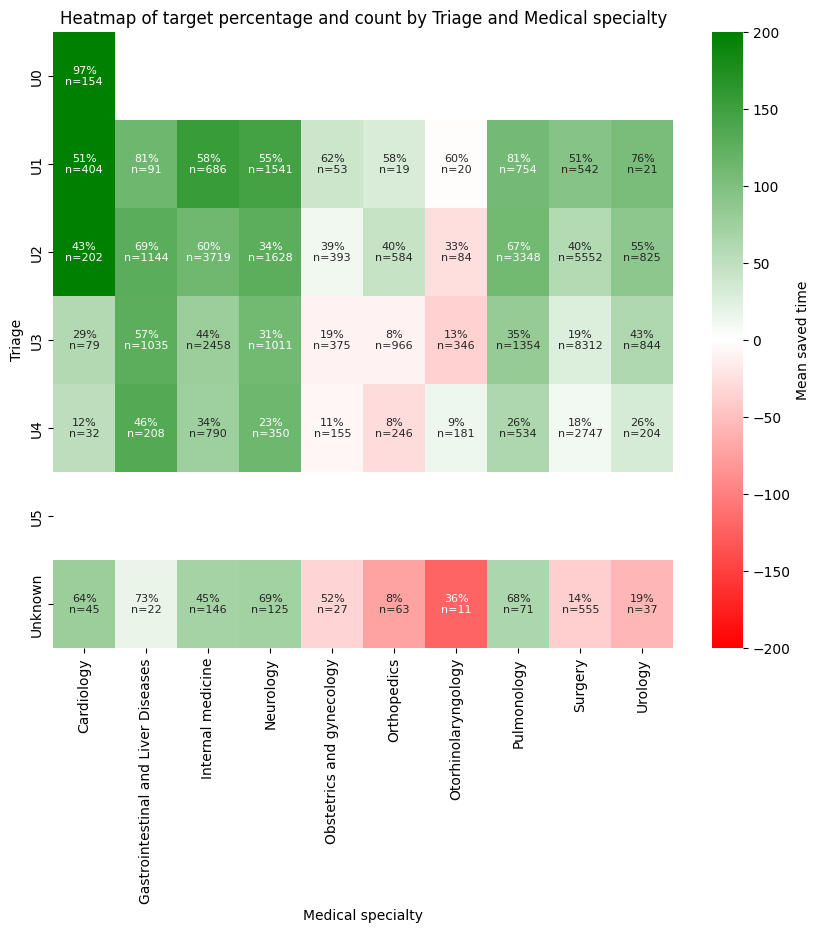


Figure S1, Saved time per patient between Medical specialty and Triage

#### Medical specialty vs Age

A strong correlation can be observed for the specialties Cardiology, Gastroenterology, and Pulmonology with the age category 88+, see Figure S2. The greatest time savings are identified for Cardiology within the age categories 78-87 and 88+. In contrast, a weaker association is found between Orthopedics and age.


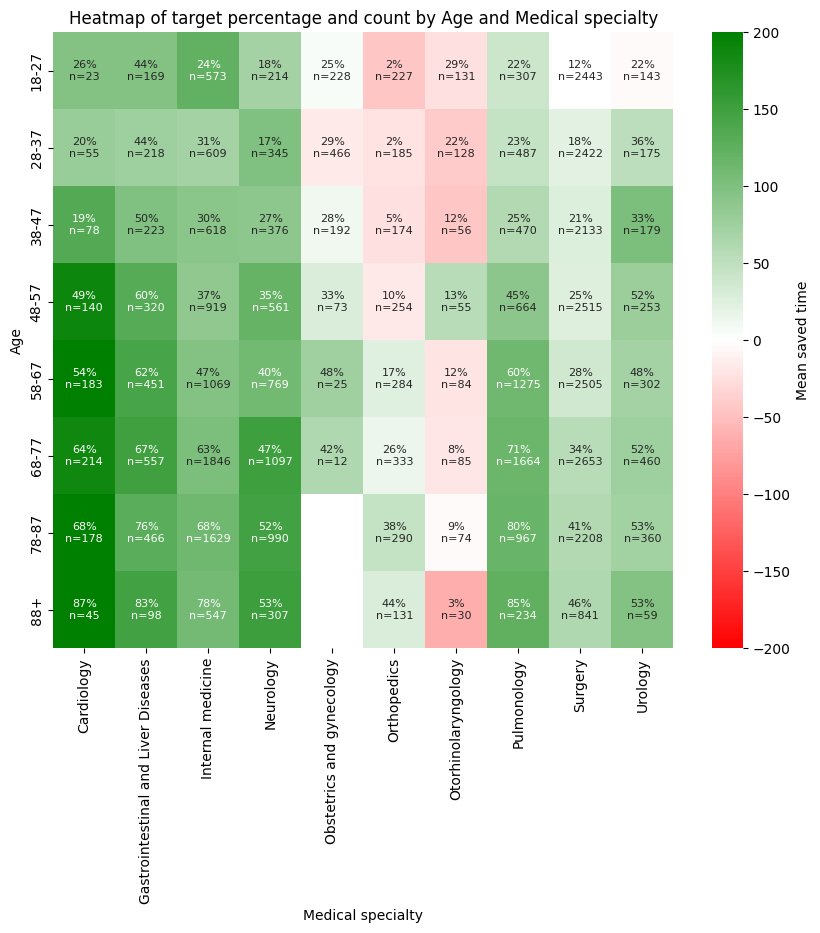


Figure S2, Saved time per patient between Medical specialty and Age

#### Medical Specialism vs Part of the day

Figure S3 showns no strong correlation between the two categories. The greatsest time savings are observed for Cardiologie during night and afternoon periods.


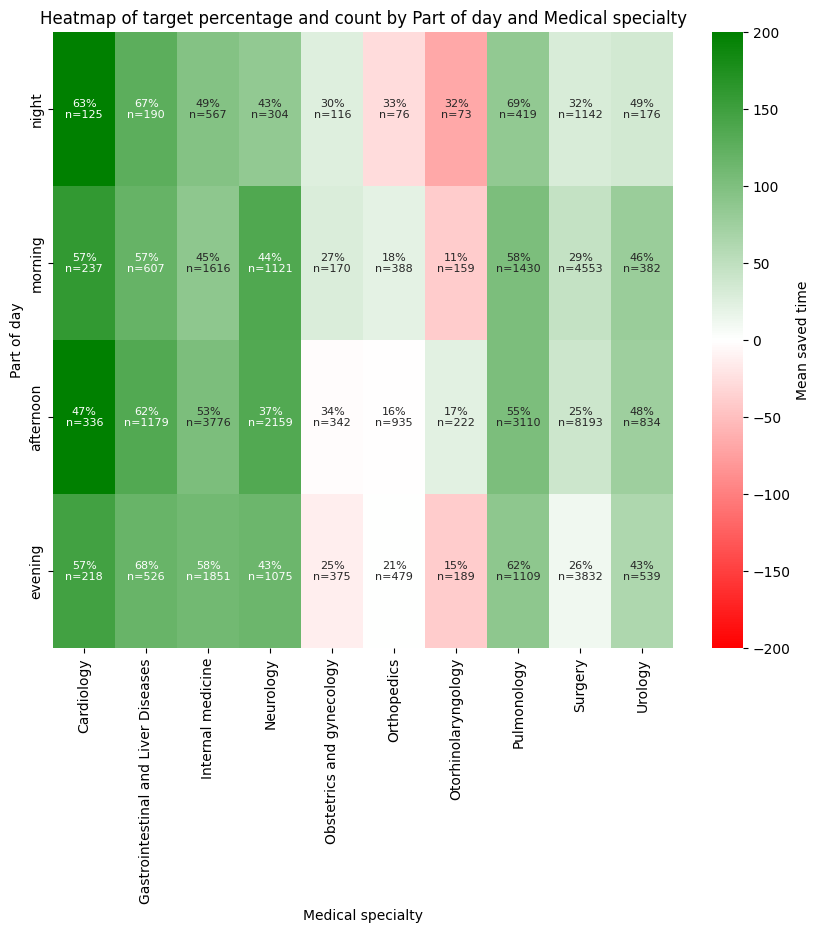


Figure S3, Saved time per patient between Medical specialty and Part of day

#### Triage vs Age

Figure S4 illustrates a strong association between U0 and all age categories, with subcategories 48-57 and 88+ contributing significantly to time savings. In contrast, weaker associations are observed between higher triage categories and age, resulting in reduced time savings achieved by the model.


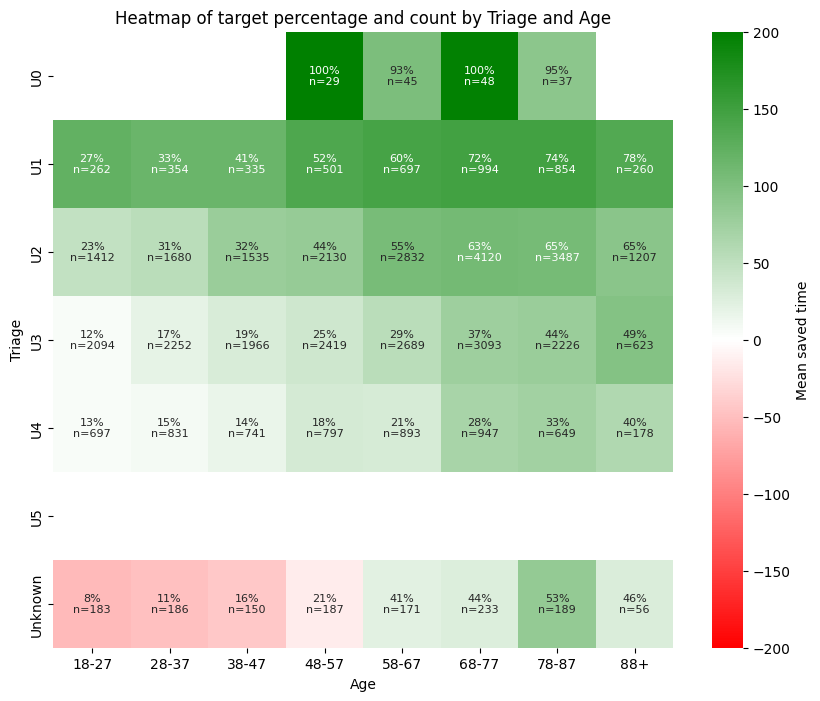


Figure S4, Saved time per patient between Triage and Age

#### Triage vs Part of the day

The heatmap in Figure S5 reveals that the triage category U0 exhibits a strong correlation with the four different times of the day. The most significant time savings are observed during the morning and evening periods. In contrast, limited associations are evident between the other subcategories in Figure S5 .


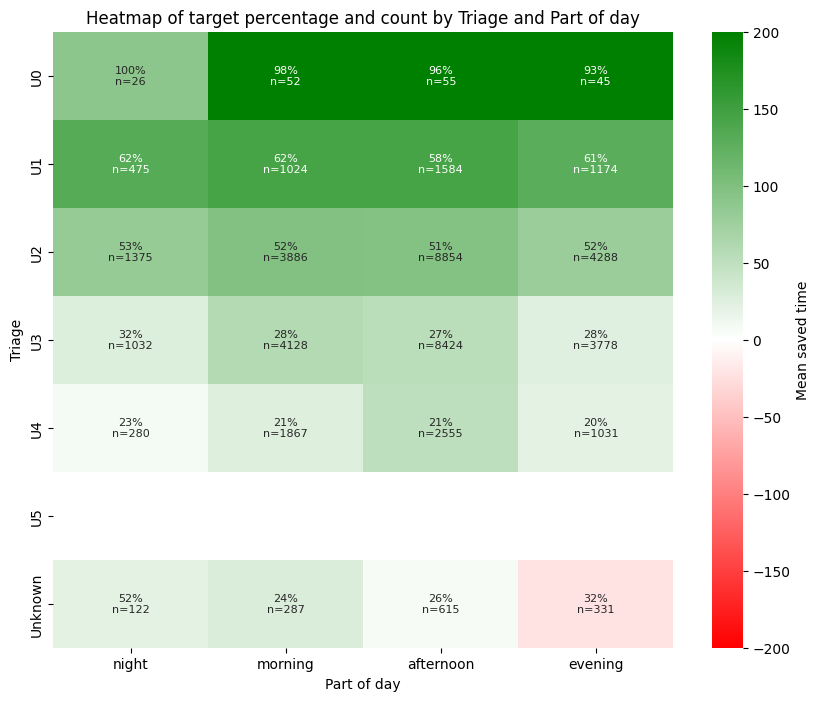


Figure S5, Saved time per patient between Triage and Part of day

#### Age vs Part of the day

Figure S6 shows no strong association between the subcategories Age and Part of the day. However, it can be observed that for ages between 58 and 88+, the mean saved time frequently exceeds 80 minutes.


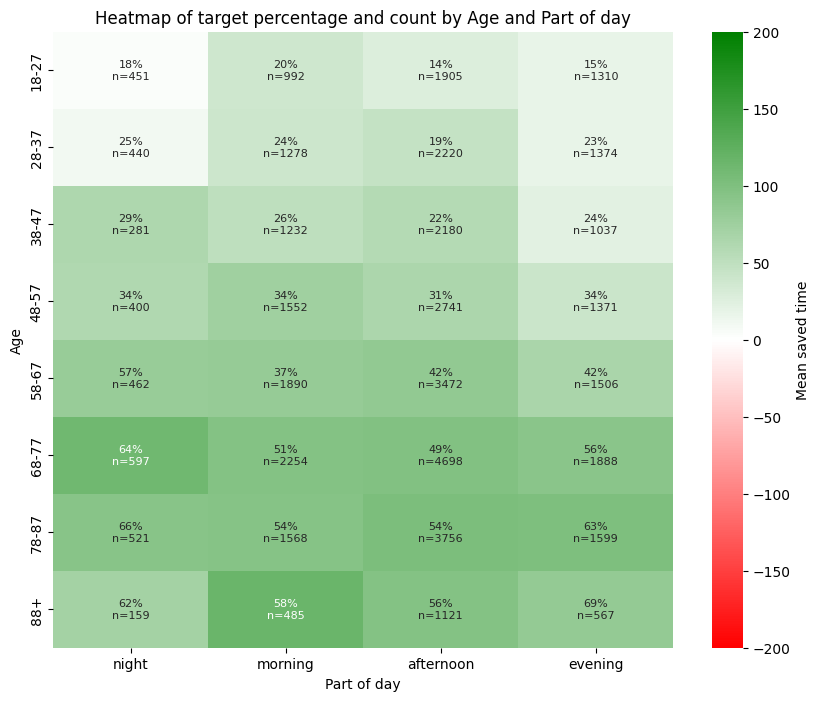


Figure S6, Saved time per patient between Age and Part of day.
