# Supplementary material for: Evaluating an AI Decision Support System for the Emergency Department: Retrospective Study
Source: JMIR AI. 2026 Jan 26;5:e80448. doi: 10.2196/80448 (PMC12887564; doi:10.2196/80448)
Supplement: Multimedia Appendix 4 [file ai_v5i1e80448_app4.docx]

Multimedia appendix 4 - CREMLS (Consolidated Reporting Guidelines for Prognostic and Diagnostic Machine Learning Models) checklist

| Item number | Item | Chapter manuscript or reasoning |
| --- | --- | --- |
| Study details |  |  |
| 1.1 | The medical or clinical task of interest | Introduction |
| 1.2 | The research question | Introduction |
| 1.3 | Current medical or clinical practice | Introduction |
| 1.4 | The known predictors and confounders of what is being predicted | Introduction and Method |
| 1.5 | The overall study design | Method |
| 1.6 | The medical institutional settings | Method |
| 1.7 | The target patient population | Method |
| 1.8 | The intended use of the ML model | Introduction and Method |
| 1.9 | Existing model performance benchmarks for this task | Introduction and Discussion |
| 1.10 | Ethical and other regulatory approvals obtained | Method |
| The data |  |  |
| 2.1 | Inclusion or exclusion criteria for the patient cohort | Method |
| 2.2 | Methods of data collection | Method |
| 2.3 | Bias introduced due to the method of data collection used | Discussion |
| 2.4 | Data characteristics | Result and Appendix I |
| 2.5 | Methods of data transformation and preprocessing applied | Appendix I |
| 2.6 | Known quality issues with the data | Discussion |
| 2.7 | Sample size calculation | Not applicable |
| 2.8 | Data availability | Data cannot be shared due to ethical reasoning. |
| Methodology |  |  |
| 3.1 | Strategies for handling missing data | Appendix I |
| 3.2 | Strategies for addressing class imbalance | Appendix I |
| 3.3 | Strategies for reducing dimensionality of data | Not applicable |
| 3.4 | Strategies for handling outliers | Not applicable |
| 3.5 | Strategies for data augmentation | Not applicable |
| 3.6 | Strategies for model pretraining | Not applicable |
| 3.7 | The rationale for selecting the ML algorithm | Method |
| 3.8 | The method of evaluating model performance during training | Method |
| 3.9 | The method used for hyperparameter tuning | Appendix 1 |
| 3.10 | Model’s output adjustments | Method |
| Evaluation |  |  |
| 4.1 | Performance metrics used to evaluate the model | Result |
| 4.2 | The cost or consequence of errors | Introduction and Discussion |
| 4.3 | The results of internal validation | Result |
| 4.4 | The final model hyperparameters | Result |
| 4.5 | Model evaluation on an external data set | Method |
| 4.6 | Characteristics relevant for detecting data shift and drift | Not applicable |
| Explainability & transparency |  |  |
| 5.1 | The most important features and how they relate to the outcomes | Result, Appendix 2, and Appendix 3 |
| 5.2 | Plausibility of model outputs | Discussion |
| 5.3 | Interpretation of a model's results by an end user | Discussion |
